# Software for Apportionment of Asbestos-Related Mesotheliomas

**DOI:** 10.1155/2016/5340676

**Published:** 2016-05-18

**Authors:** Robert M. Ross

**Affiliations:** Pulmonary and Critical Care Medicine, Baylor College of Medicine, Houston, TX 77030, USA

## Abstract

Patients with an asbestos-related mesothelioma may be legally entitled to financial compensation. In this context, a physician may be called upon to apportion the contribution of an asbestos containing product or facility where there was asbestos exposure in the development of that individual's mesothelioma. This task is mathematically not simple. It is a complex function of each and the entire individual's above-background asbestos exposures. Factors to be considered for each of these exposures are the amount of exposure to mesotheliogenic fibers, each of the asbestos containing products' potency to cause mesothelioma, and the time period when the exposures occurred relative to when the mesothelioma was diagnosed. In this paper, the known factors related to asbestos-related mesothelioma risk are briefly reviewed and the software that is downloadable and fully functional in a Windows® environment is also provided. This software allows for rapid assessment of relative contributions and deals with the somewhat tedious mathematical calculations. With this software and a reasonable occupational history, if it is decided that the mesothelioma was due to above-background asbestos exposure, the contribution of an asbestos containing product or a time period of asbestos exposure can be apportioned.

## 1. Introduction

When a physician is confronted with a mesothelioma it is important to determine whether it is asbestos-related. If it is, the patient may be entitled to financial compensation. However, it should be remembered that not all cases of mesothelioma are asbestos-related.

There is a background rate of mesothelioma unrelated to asbestos exposure. Based on Surveillance Epidemiology and End Results (SEER) data, people below about age 50, who would be unlikely to have been exposed to much asbestos in a work or household contact situation, have a background rate of about 1 per million for both males and females [1]. J. C. McDonald and A. D. McDonald also determined a similar background rate of about 1-2 per million per year [2].

Other non-asbestos-related risk factors for mesothelioma include chronic inflammation such as from tuberculosis (or its complications) or empyema [3]. Radiation for malignancy and possible malignancy itself, or its treatment, also increase the risk of developing mesothelioma [4]. Simian-40 virus (SV 40) has also been suggested by some as a possible factor, although more recent data suggests that it may not [5]. Environmental and genetic factors also play a role. For example, it is well known that there is an increased prevalence of mesothelioma in parts of Turkey where environmental erionite is present and possibly in the United States as well [6]. Further, BAP1 germline mutations have recently been found to be associated with mesothelioma [7]. A relatively recent review discusses many of the issues involved in the pathogenesis of malignant mesothelioma and the role of environmental and genetic factors [4].

However, asbestos exposure is the most common cause of mesothelioma, particularly in men. A French study found that the attributable-risk fraction for mesothelioma from occupational exposure to asbestos in men was 83.2% (95% CI 76.8–89.6) [8].

At present, a reasonable estimate is that about 80% of mesotheliomas in men and about 40% in women are associated with occupational exposure to asbestos [9]. However, it should be remembered that the exact proportion of asbestos-related mesotheliomas will vary with the population studied and is different for pleural versus peritoneal mesotheliomas. The determination of what represents an asbestos-related mesothelioma also has certain inherent uncertainties as not all people exposed to asbestos have asbestos-related findings and all people have asbestos fibers in their bodies. Further, the proportion of asbestos-related mesotheliomas will change over time. It has been about 40 years since the government has legislated limited asbestos exposure. Trends suggest that mesothelioma rates have peaked. As the total declines due to a reduced number of asbestos-related mesotheliomas, the number of background cases and cases due to factors other than asbestos will become a greater percentage of the total.

## 2. Apportionment

If, after careful evaluation, it is concluded that the mesothelioma is asbestos-related, particularly for legal purposes, apportionment may be desirable. Many of these people have been exposed to asbestos in a host of different ways at different times. Often, a physician or another expert will be requested to give an estimate of the relative contribution of a certain product, or time period at a particular facility, to the individual's mesothelioma. However, determining the relative contribution, or “mesotheliogenic potential or risk,” of any product or time period of exposure is not simply a matter of relative exposure dose. It is a complex computation of all of the exposures involving the time periods when the exposures occurred, the concentration of the asbestos during each of the time periods, and the potencies of the asbestos fibers to cause mesothelioma during each exposure period, that is, the mesotheliogenic potential of a particular product or time period, relative to the total mesotheliogenic potential of the entire person's above background asbestos exposures.

The assessment requires a reasonable understanding of the individual's asbestos exposure history. It also requires somewhat laborious mathematical calculations. This paper discusses the data presently believed to be required to make the appropriate calculations and the scientific basis for the computations. Also provided is a fully functioning software program that makes the calculations and provides the apportioned results.

## 3. Asbestos-Related Mesotheliogenic Potential

When utilizing this software for apportioning an individual mesothelioma, it is assumed that all exposures above background contributed to the mesothelioma in proportion to their “mesotheliogenic potential or risk.” The “mesotheliogenic potential or risk” of any exposure period or asbestos containing product depends upon many factors, including fiber type, fiber dimension, the time period since exposure, and the total respirable mesotheliogenic asbestos fiber dose during each time period as well as all other above background asbestos exposures. As previously mentioned, genetic factors also seem to play a role [7, 10]. However, genetic factors are not considered in apportionment for an individual mesothelioma as, until shown otherwise, they are assumed to affect all risk factors equally and so cancel out mathematically.

In 2003, the EPA published a Final Draft regarding a protocol to assess potential human health risks associated with exposure to asbestos. The risks were determined by reviewing exposure-response coefficients estimated for asbestos from approximately 150 epidemiological studies, for which approximately 35 contained exposure data sufficient to derive a quantitative exposure/response relationship.

Some of the conclusions of the Final Draft included that fibers longer than 10 microns and thinner than 0.4 microns were considered pathogenic, amphiboles were orders of magnitude more potent than commercial chrysotile for the development of mesothelioma, and the possibility that pure chrysotile did not cause mesothelioma in humans could not be ruled out by the available data [11]. These issues will now be briefly discussed.

## 4. Fiber Length

Asbestos is an inert fibrous rock. It is highly resistant to heat, chemical reactions, and degradation. These features made it extremely useful in industry. Under certain conditions asbestos has the potential to cause disease after it is breathed into the body. Presently, most authorities believe that only certain sizes and shapes of asbestos have the combined ability to be inspired deep into the lung (terminal bronchioles and alveoli) and cause disease. They must be relatively long and thin [12, 13].

Based on findings from epidemiology studies, laboratory animal studies and in vitro genotoxicity studies, combined with the lung's ability to clear short fibers, there is a strong weight of evidence that asbestos fibers shorter than 5 microns are unlikely to cause cancer in humans [14].

A meta-analysis was performed that addressed mineral type and fiber size on mesothelioma risk. The study did not find any consistent evidence for any potency for fibers <5 microns in the development of mesothelioma [15].

The pathogenicity associated with different length fibers was also recently studied using nanofibers which can be engineered to be much more homogeneous in terms of fiber length than natural asbestos fibers. In animal experimental studies, using a very specific panel of different length classes, otherwise identical in composition, diameter, and solubility, a clear threshold effect was found demonstrating that fibers 5 microns and greater were pathogenic to the pleura while shorter fibers were not [16].

## 5. Time Period since Exposure

The risk of developing mesothelioma has been found to increase as a power function of time after exposure [17]. The power function is about 3. Since there is almost no risk of developing mesothelioma during the first 10 years after exposure, the time period is usually lagged by 10 years. It has been approximated by time since first exposure lagged by 10 years and then squared [18].

A more recent review also found that over time mesothelioma rate was well predicted by time since first exposure (lagged by 10 years) squared. They also pointed out this produced very similar results to time since first exposure (not lagged) cubed [19].

It is possible that, at least for pleural mesotheliomas, the rate may not increase monotonically with time since first exposure. A relatively large cohort study where workers were followed for 50 years found that the rate increased for the first 40–45 years of time since first exposure and then seemed to plateau. This pattern was not seen for peritoneal mesotheliomas. The authors suggested that the risk for pleural mesothelioma, rather that showing an indefinite increase, might reach a plateau when a sufficiently long time had elapsed since exposure [20].

In summary, for similar fiber types, earlier exposures to asbestos have a dramatically greater effect on the risk of developing mesothelioma than exposures obtained later. The software program assumes that similar type and dose of asbestos exposure occurring twice as far in the past (lagged by 10 years) would have a fourfold greater effect on mesothelioma risk.

## 6. Fiber Dose

There is a threshold for exposure to asbestos before there is a doubling of the background risk of developing mesothelioma [21]. The threshold dose depends on the type of asbestos fiber. However, above the threshold, the likelihood of developing mesothelioma increases with increasing exposure dose.

The most common asbestos exposure has been to commercial chrysotile, which usually has some amphibole contamination (<10%). The literature suggests that cumulative exposure to commercial chrysotile in the range of 15–500 fiber/cc years is required to statistically significantly increase the risk for mesothelioma [22]. There may also be some difference if the chrysotile exposure is continuous versus episodic [23]. The minimal dose associated with exposure to amphibole asbestos is much lower [24].

The finding of a threshold suggests that not only dose but concentration may also play a role in the development of a mesothelioma. There is biological support for this idea as high concentrations can overwhelm defense mechanisms and so produce a greater risk than a similar dose at a lower concentration [25]. A review of the epidemiological studies also suggests that the effect of exposure concentration may be supralinear [24].

However, as yet there is not a specific exposure above background that can be definitively shown not to contribute any risk. Therefore, when an individual has an asbestos-related mesothelioma, the software assumes that all above background asbestos exposures contribute in proportion to their mesotheliogenic potential. The operator determines what potency factor to give for any specific exposure. The effect of increasing dose during any time period is considered to increase the risk of developing a mesothelioma in a linear fashion. That is, the present approach is to use all exposures above background and, assuming exposure to similar types of asbestos over any given year, the program assumes a linear relationship between the respirable exposure dose and the risk of developing a mesothelioma.

## 7. Fiber Type

Commercially used asbestos comes in two types, serpentine asbestos (chrysotile) and amphibole asbestos, consisting primarily of crocidolite and amosite, although other amphiboles have also been used. Chrysotile is a sheet silicate that rolls into nanosized tubular structures possessing a hollow core. Amphiboles are chain silicates. Chrysotile is the most common, commercially used form of asbestos. Historically, it represents approximately 90–95% of all asbestos used in the United States.

As previously discussed, most of this chrysotile asbestos is not pure. It also contains small amounts of the amphibole tremolite or other amphiboles. That is, most workers that have been exposed to commercial chrysotile asbestos have been exposed to chrysotile associated with small amounts of amphiboles.

Present scientific knowledge indicates that the relative risk of developing mesothelioma varies significantly among the different types of asbestos. This is felt to be because amphiboles have much greater biopersistence than chrysotile.

In 1960, Wagner et al. found a marked increase in mesotheliomas in a crocidolite (an amphibole) mining area of South Africa [26]. Since then, there has been extensive study of the relationship between asbestos type and mesothelioma.

In 1999, Rees et al. performed a case control study of mesotheliomas in South Africa. They reviewed 123 cases of mesothelioma and found that none had pure chrysotile exposure [27].

In 2000, Hodgson and Darnton reviewed the literature in an attempt to determine the lifetime risk for the development of a mesothelioma associated with exposure to chrysotile, often with small amounts of tremolite (commercial chrysotile), and the amphiboles, amosite, and crocidolite. Overall, they found that the specific risks for mesothelioma were broadly in the ratio of 1 : 100 : 500 for commercial chrysotile, amosite, and crocidolite, respectively. For occupational-type exposures, the ratio was about 1 : 35 : 200 [24].

Berman and Crump also utilized a meta-analysis to address mineral type and fiber size on mesothelioma risk. 12 locations were included in their meta-analysis. They found that the data strongly indicated that amphiboles are much more potent than commercial chrysotile for the development of mesothelioma. The potency difference varied with different studies but overall was in the order of 100-fold higher value. Further, they could not find convincing evidence that pure chrysotile (without amphibole contamination) could cause mesothelioma in humans [19].

In summary, the weight of present evidence is that pure chrysotile is probably not mesotheliogenic in humans. Commercial chrysotile which is contaminated with small amounts of amphibole is mesotheliogenic but of relative low potency while amphiboles are highly potent. The software program requires the operator to input the relative potencies for the types of asbestos that the person was exposed to.

## 8. Risk Apportionment for an Asbestos-Related Mesothelioma

When an individual develops a mesothelioma that is due to asbestos exposure, it is often useful to determine the relative contribution of different asbestos-containing products, or different work locations in its development.

To answer the question regarding the relative contribution of an asbestos-containing product, or a time period of exposure to asbestos in the development of an asbestos-related mesothelioma, it would be beneficial to have estimates of the cumulative respirable exposures of mesotheliogenic fibers of the various types of asbestos that the individual was exposed to, as well as when the exposures occurred. Usually it is very difficult to retrospectively determine specific individual exposures. However, various documents and articles provide job exposure indices or give information about fiber types and exposure dose ranges for various tasks and time periods [28, 29].

Fortunately, a reasonably accurate assessment of the contribution of a product or a time period in the development of an individual's asbestos-related mesothelioma can often be made without having detailed specific exposure information. This is because, in a given individual who has an asbestos-related mesothelioma, only relative exposure doses and potencies are needed when assigning relative contribution. Absolute values are unnecessary. For example, if an individual did similar type of work at different facilities over several years, the same dose and potency values can be used for each of the years. Only the time period at each facility would be different. With this information apportionment can be accurately determined. It does not matter what values for dose and potency are used unless the individual was exposed to asbestos of a different type or dose elsewhere. However, even if there are any other exposures, if these exposures are entered as appropriate multiples of the initial values chosen the apportionment results will be meaningful. The absolute values do not matter as they cancel out mathematically.

## 9. Software for Apportionment

Accompanying this paper, in the Supplementary Materials available online at http://dx.doi.org/10.1155/2016/5340676 is a folder named “MesoContribution.” This is fully functional, downloadable software that can be utilized in a Windows environment, to perform the mathematical calculations necessary to apportion the contribution of any product or time period of exposure to the mesothelioma.

If your computer does not have Microsoft runtime Access® already installed, you must install it before the program will run. You can download it from the Microsoft Web site. Presently, the link is http://r.office.microsoft.com/r/rlidAccessRuntime. Follow this link and download the runtime for Access. Alternatively, you can go to Microsoft's web site and download the Access runtime from there. Save it to your hard disk and then run it by double-clicking on “AccessRuntime.exe” and follow the directions. When you run it on your computer you will be asked to put in a “name” and “company”. Enter anything and then pick “typical” installation.

Once you have Access installed you are ready to install the program. First, you must download the program folder (MesoContribution) to your computer. You can save it to any folder you desire or the desktop. It is a “zip” folder. “Unzip” the folder by “right clicking” and use “extract all files.” This will produce a subfolder called “MesoContribution.” Then, go to the folder named “MesoContribution.” Inside the folder, there will be a folder called “files” as well as 2 files, one called “setup” (or setup.exe) and the other called “autorun” (or autorun.inf). Double click on “setup.” An Access program named “MESOContribution” will be placed on your desktop.

## 10. Utilizing the Software

To utilize the software on your computer go to the desktop and open the Access program “MESOContribution.” There will be an entry screen with multiple fields. There is also a button with “Instructions” and an “Example” button.

Enter the year the mesothelioma occurred in and the number of months of any years when the person was exposed to asbestos above background levels. It is not necessary to enter any time periods when there was no exposure to asbestos above background. If exposure to more than 1 asbestos containing product occurred during the same month or months of a given year, the time period may be duplicated with appropriate adjustments for concentration and potency of each product. The same can be done for different work places during similar time periods.

As well as the number of months, the relative exposure concentration and relative potency for causing mesothelioma must be entered. To do this, enter the average 8-hour time weighted average during the time period chosen. Sometimes exposure data may be obtained as dose (fibers per cc years) of various products or work place situations during certain years. In that case, for each exposure, enter the dose value in the “concentration” field and 12 in the “months” field for that particular year.

Another way to enter concentrations is to assign the lowest exposure concentration an arbitrary value of 1. Then, all other exposure concentrations can be entered as a multiple of 1. For example, if someone were exposed to an average of 0.5 fibers per cc of asbestos at one time and 2.5 fibers per cc at another, these values may be entered. However, the results would be just as accurate if the dose were entered as 1 and 5, respectively, as long as the relationship is kept constant for all entries.

A similar relationship holds true for potency. Decide on the potency ratio of commercial amphiboles to commercial chrysotile. For example, a reasonable estimate might be about 50 : 1. Commercial chrysotile would then be given a potency of 1. If a worker worked with pure amphibole, the potency would be 50. If he worked only with commercial chrysotile, the potency would be 1. If he worked in an area where he was exposed to all types of asbestos products it would be likely that he would be exposed to chrysotile and amphiboles in proportion to their usage. Since about 90% of asbestos used was chrysotile, the average potency would be about 90% of 1 plus 10% of 50 or about 6.

If the individual was exposed to similar concentrations for similar time periods over multiple years, the program allows you to enter the values and the final year and multiple computations will be made.

The program also allows you to add a “description” for each line entry and a “group description” if desired. For example, all exposures of a particular product may be grouped under the product's name in “group description” and each of the places or work situations can be entered as a “description.” However, these entries are optional.

There is also a field at the bottom of the entry screen called “max years” that allows you to decide on a time since first exposure (such as 40 years), lagged by 10 years, after which the risk of mesothelioma related to time plateaus.

After these values are entered the program will calculate the contribution of each entry to the total mesotheliogenic risk. Each entry's absolute mesotheliogenic risk is the product of exposure concentration, potency value, and months/12 *∗* (year of mesothelioma diagnosis – (entry year + 10)) squared, and the percentage contribution is the product of exposure concentration, potency value, and months/12 *∗* (year of mesothelioma diagnosis – (entry year + 10)) squared divided by the sum of all entry contributions times 100.

The program also provides a printout of each entry's absolute mesotheliogenic risk value, based on the values entered, and its percentage contribution to that individual's asbestos-related mesothelioma. The report will display the results grouped and subtotaled in terms of percentage contribution by “group description.”

## 11. An Apportionment Example

**Figure 1:**
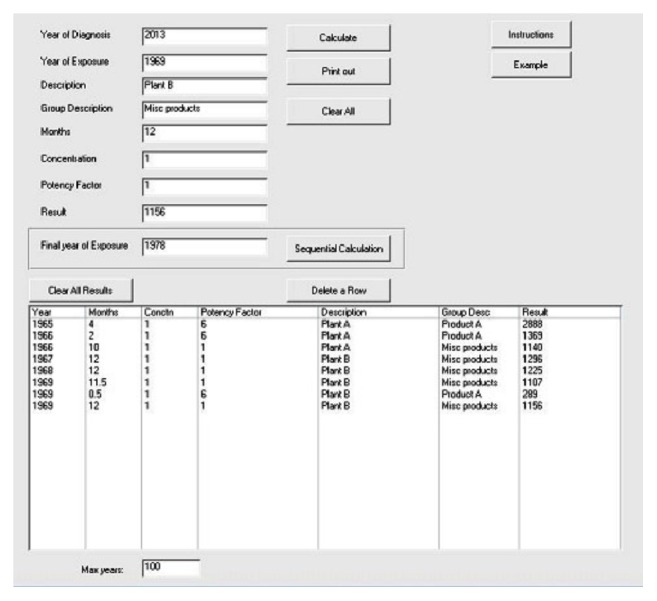
Data input screen. Please see text for description of input.

Figure 1 provides an example input. In this hypothetical case a person who was found to have an asbestos-related mesothelioma in 2013 had worked with a particular asbestos containing product “Product A” in 1965 for 4 months and 1966 for 2 months at “Plant A.” The estimated exposures there were “1” and the potency of the product was estimated at “6” as it was about 90% chrysotile and 10% amphibole. He did not work with this product elsewhere but did work around other asbestos containing materials from 1966 to 1970. The exposure was relatively similar as he did a similar job and so was given a concentration of “1” also. However, there was no significant amphibole exposure so the potency was estimated as “1.” As well as his regular work, in 1969 he also again worked with “Product A” for 2 weeks. He estimated that the exposure conditions of that event were similar to his exposures to “Product A” in 1965 and 1966.

Table 1 provides the printout. In this case, asbestos containing “Product A” would have contributed approximately 44% of the apportionment to the mesothelioma.

All exposures are listed, grouped by “group description.” Provided are the year of exposure, number of months, average exposure concentration during that time period, and the mesotheliogenic potency of the asbestos that the person was exposed to as well as descriptive information. The program calculates and displays the “result,” which is the absolute mesothelioma risk, and the relative contribution to the mesothelioma as the “percent” contribution.

## 12. Limitations

The program does not determine if the mesothelioma is asbestos-related. It only takes care of the somewhat cumbersome mathematics required. Further, appropriate values to enter are the responsibility of the operator. The program does not determine what the actual respirable exposures were or what the correct relative potencies of the various asbestos containing products were. The results are only as accurate as the input. The program utilizes the present scientific assessment of the factors affecting mesothelioma risk. In the future, other factors (such as a threshold concentration) may be found to also play a role. Further, an individual's memory of different exposures can significantly affect the results. However, as only relative exposures and potencies are important and not absolute values, many errors will cancel out mathematically and so usually a reasonable estimate of contribution can be determined and apportionment assessed.

## 13. Conclusions

Software that can be of assistance in dealing with many medical issues will be an important component of medicine in the future. However, at the present time there are very few software programs provided through peer reviewed medical journals [30]. This paper discusses some of the issues related to the present level of knowledge regarding the factors that contribute to the mesotheliogenic risk of developing an asbestos-related mesothelioma. Software is provided to perform the necessary calculations. When an individual has an asbestos-related mesothelioma, this software can be used to apportion the contribution of any particular asbestos containing product, work place, or time period of asbestos exposure to its development.

## Supplementary Material

The Supplementary Material consists of the program “Mesocontribution”. It is a program written in Microsoft Access and runs in Microsoft Windows. Please see “Section 9. Software for Apportionment”, for instructions regarding how to install and run it on your computer.

## Figures and Tables

**Table 1:** Print out. Mesothelioma year diagnosis: 2013.

Year	Months	Concentration	Potency factor	Description	Group description	Result	Percent
Group description: misc. products							
1966	10	1	1			1140	10.96%
1967	12	1	1			1296	12.46%
1968	12	1	1			1225	11.78%
1969	11.5	1	1			1107	10.64%
1970	12	1	1			1089	10.47%

						5857	56.30%43.70%X

Group description: product A							
1965	4	1	6	Plant A	Product A	2888	27.76%
1966	2	1	6	Plant A	Product A	1369	13.16%
1969	0.5	1	6		Product A	289	2.78%

						4546	43.70%43.70%X

						10403	100.00%

## Additional Points

The program “MESOContribution” is written in Microsoft Access. This program can be downloaded using the link provided in Section 9. It is a “zip” folder, which is then extracted on your personal computer. The program runs in a Microsoft Windows environment. Use of the program does not require a license. The program requires the Access runtime available from Microsoft. Use of Access runtime is subject to Microsoft's licensing. Access and Microsoft are registered trade names of Microsoft Corporation, Redmond, Washington.
